# Biomass loss and composition change of energycane and biomass sorghum during aerobic and anaerobic storage

**DOI:** 10.1038/s41598-025-34190-1

**Published:** 2026-01-03

**Authors:** Yubin Yang, Tanumoy Bera, Lloyd T. Wilson, Fugen Dou, John L. Jifon, William L. Rooney, Hamid Araji, Jesse I. Morrison, Brian S. Baldwin, Joseph E. Knoll, Alan L. Wright, Dennis C. Odero, Hardev S. Sandhu, Anna L. Hale, Himaya P. Mula-Michel, Jing Wang

**Affiliations:** 1 Texas A&M AgriLife Research and Extension Center, 1509 Aggie Drive, Beaumont, TX 77713 USA; 2 Texas A&M University and Texas A&M AgriLife Research and Extension Center, 1509 Aggie Drive, Beaumont, TX 77713 USA; 3 Texas A&M AgriLife Research and Extension Center, 2415 E Hwy 83, Weslaco, TX 78596 USA; 4 Department of Soil and Crop Sciences, Texas A&M University, College Station, TX 77843 USA; 5 Department of Plant and Soil Sciences, Mississippi State University, Mississippi State, MS 39762 USA; 6 USDA-ARS, Crop Genetics and Breeding Research Unit, 115 Coastal Way, Tifton, GA 31793 USA; 7 Indian River Research and Education Center, University of Florida, 2199 South Rock Road, Fort Pierce, FL 34945 USA; 8 Everglades Research and Education Center, University of Florida, 3200 East Palm Beach Road, Belle Glade, FL 33430 USA; 9 USDA-ARS, Sugarcane Research Unit, 5883 USDA Road, Houma, LA 70360 USA; 10 Department of Ecosystem Science & Management, University of Wyoming, Laramie, WY 82071 USA

**Keywords:** Energycane, Biomass sorghum, Biomass loss, Moisture content, Composition change, Storage duration, Biofuels, Bioenergy

## Abstract

Energycane and biomass sorghum are two of the most promising cellulosic energy crops in the southeastern US. This study aims to determine the biomass loss and composition change of energycane and biomass sorghum in storage under different environments. Aerobic and anaerobic storage trials were conducted in seven locations across the southeastern US for energycane and six locations for biomass sorghum for 3, 6 and 9 months. Results revealed that crop type accounted for less than 3% of the variability in moisture content and biomass loss, suggesting similar storage characteristics between energycane and biomass sorghum. Average moisture content decreased from 60.7 to 50.4% after 9 months in covered aerobic storage piles but increased from 62.9 to 67.2% for anaerobic storage in anaerobic silage bags. Dry matter loss averaged 49.9% after 9 months of aerobic storage and 40.3% for anaerobic storage. Dry matter loss was described as a non-linear function of storage duration and average storage moisture, with greater loss for longer duration and higher storage moisture. Average changes in biomass composition for cellulose, hemicellulose, lignin, and ash were all less than 3% during the 9-month storage regardless of storage types. Results from this study provide valuable insights on changes in biomass quantity and quality during storage and fill a critical knowledge gap in addressing the challenge of year-round biomass supply to biorefineries.

## Introduction

Energycane (*Saccharum* spp.) and biomass sorghum (*Sorghum bicolor*) are two promising cellulosic energy crops in the southeastern US^1^. Energycane are interspecific hybrids derived from crosses between *Saccharum officinarum* and *S. spontaneum.* They tend to have greater cold tolerance, higher fiber, and lower sugar contents compared to commercial sugarcane^2,3^. Biomass sorghum are photoperiod-sensitive hybrids that have drought tolerance and high water-use efficiency^4^. Both energycane and biomass sorghum are warm-season crops. In the southeastern US, energycane is usually harvested from October through December while biomass sorghum is usually harvested from September through October.

Research on these two energy crops has mostly focused on yield at peak biomass^4,5^. Several studies have focused on seasonal growth dynamics of biomass sorghum^6,7^ and energycane^8^. Biomass storage has emerged as a crucial upstream challenge in cellulosic biofuels development^9^. Just-in-time harvest and extended storage can potentially address the challenge of year-round biomass supply for biorefineries and spread harvesting operations over an extended window. However, questions on early harvest yield penalty, post-maturity standing biomass loss, and composition change during storage, as well as impacts of early and late harvest on regrowth of energycane have not been adequately addressed. Due to the seasonal nature of biomass dynamics, harvest either pre- or post-peak biomass will incur a yield penalty^8^ or post-maturity yield loss^10^. Long-term storage could further extend the biomass supply window, enabling biorefineries to operate year-round with a consistent feedstock supply despite seasonal variation in biomass yield^11^.

Strategies for traditional on-farm forage storage can be broadly categorized into aerobic dry storage and anaerobic wet storage^12^. Dry storage reduces the availability of biological water, which inactivates cellulose-degrading fungi and bacteria. Since these organisms can be revived upon re-wetting, dry materials must remain dry to maintain stability. Wet storage relies on oxygen limitation and the action of acid-producing fermentative microorganisms to reduce the pH (< 4.5) and inhibit the growth of other microorganisms that degrade cellulose^12^.

The most common dry storage options are round bale and square bale storage. Although round bale storage has been proven successful for thin-stem grass feedstocks such as switchgrass (*Panicum virgatum*), agricultural residues such as thick-stem stover of corn (*Zea mays* L.) have proven to be more challenging^13^. In a round bale format, corn stover does not form a tight thatch and will not reliably shed water like comparable thin-stem grass feedstock bales^13^. Square bale storage can serve as an alternative^14^, but potentially at greater biomass loss due to less ability to shed precipitation when uncovered^15,16^.

Dry bale storage is feasible only in areas of the country where harvest conditions on soil and weather permit in-field drying and collection. In the southeastern US, wet harvest conditions and environmental factors pose major challenges for dry storage. Consequently, storing high moisture biomass for bioenergy use in this region is the most likely alternative^17^. This is especially true for energycane and biomass sorghum with their thick stems and high moisture content at the time of harvest^8^.

Anaerobic wet storage systems are an alternative to dry storage and have consistently and successfully demonstrated their ability to preserve biomass in long-term storage for livestock feed and forage^11^. Wet storage systems are based on forage chopping herbaceous biomass in the field at moisture content between 40 and 65% (wet basis)^11^. Under anaerobic conditions, lactic acid bacteria convert water-soluble sugars into organic acids, resulting in a low pH (< 4.5) storage condition, thus preserving moist biomass^18^. Ensiling options include tower silos, bunker silos, and silage bales. Most studies on biomass storage were on corn stover^12,19–21^ and switchgrass^15,22–26^. Several storage studies involved forage and sweet sorghum^27^, switchgrass and reed canarygrass (*Phalaris arundinacea*)^22^, and corn stover and forage sorghum^12^. However, there are no studies on dry matter loss and quality change of energycane and biomass sorghum during storage under the hot and wet environments in the southeastern US. The objectives of this study were 1) determine the biomass loss (i.e. dry matter loss), moisture dynamics, and composition change of energycane and biomass sorghum during aerobic and anaerobic storage and 2) assess the feasibility of long-term storage in addressing the challenge of year-round biomass supply to biorefineries in the southeastern United States. Achieving these objectives will fill a major knowledge gap on biomass loss and composition change of the two feedstocks during storage in the southeastern US. It will provide critical data to develop biomass production and storage strategies that can effectively address year-round biomass supply, thus promoting biorefinery development in the region.

## Results

### Analysis of variance on biomass moisture content during storage

**Fig. 1:**
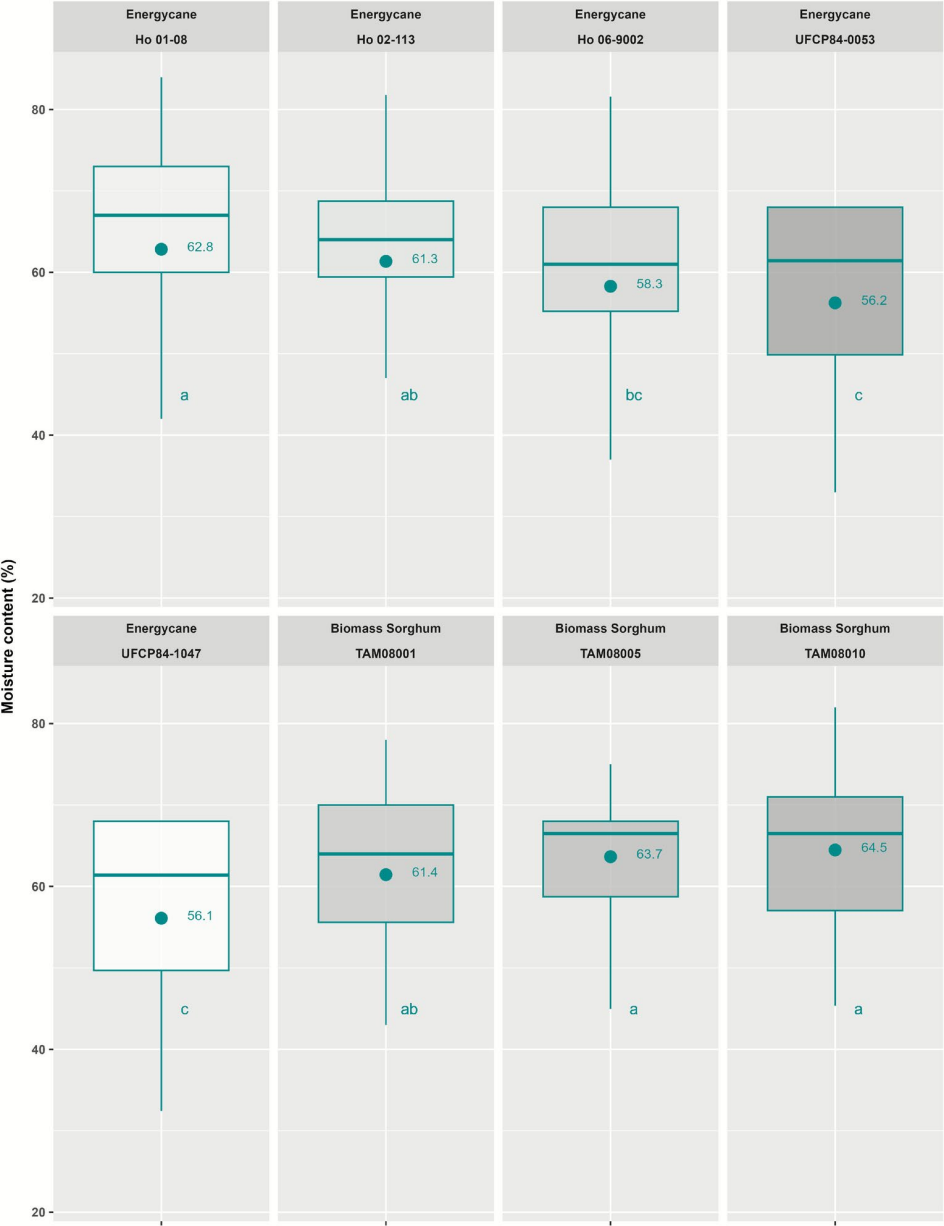
Moisture of machine-chopped biomass at the time of storage for different energycane and biomass sorghum genotypes (Combined data for 2022 and 2023) (symbol cycle ● represents mean as indicated by the number). Genotypes having the same lowercase letter are not significantly different from each other at 0.05 with Tukey’s HSD multiple comparison test.

Biomass sorghum TAM08010 had the highest moisture content of 64.5% at the start of storage, followed by TAM08005 (63.7%), energycane Ho 01–08 (62.8%), and Ho 02–113 (61.3%). Energycane UFCP84-1047 and UFCP84-0053 had the two lowest moisture contents of 56.1% and 56.2%, respectively (Fig. 1). Biomass sorghum had a mean moisture content of 63.2% at the start of storage, which was significantly higher than 60.4% for energycane (Fig. 1).

The top three main effect factors that affect biomass moisture were site, storage type, and genotype, accounting for 24.1, 5.6 and 0.8% of the total variability, respectively (Table 1). Year was not a significant factor. Although crop type had a significant effect on biomass moisture, it accounted for only 0.2% of the variability. The top three two-way interactions that affect biomass moisture were site × storage duration, site × storage type, and site × year, accounting for 9.7, 9.1 and 5.2% of the total variability, respectively (Table 1). The main effects and two-way interactions accounted for 61.8% of the total variability (Table 1). All main effects and two- and three-way interactions accounted for 81.6% of the total variability.

**Table 1:** Analysis of variance and percentage of variance explained by the main effects and two-way interactions on moisture content, biomass loss, and composition change (cellulose, hemicellulose, lignin, and ash) during biomass storage.

Main effects & two-way interactions*	Moisture			Biomass loss			DF	Cellulose		Hemicellulose		Lignin		Ash	
	DF	*P*	Var. (%)	DF	*P*	Var. (%)		*P*	Var. (%)	*P*	Var. (%)	*P*	Var. (%)	*P*	Var. (%)
Site	6	< 0.001	24.1	6	< 0.001	5.6	–	–	–	–	–	–	–	–	–
Year	1	0.006	0.0	1	< 0.001	0.7	1	0.579	0.0	< 0.001	4.9	0.004	0.9	< 0.001	7.4
Crop Type	1	< 0.001	0.2	1	< 0.001	0.1	1	< 0.001	24.8	< 0.001	25.7	< 0.001	12.7	0.026	0.9
Genotype	6	< 0.001	0.8	6	< 0.001	0.8	4	0.027	1.5	0.003	1.4	< 0.001	2.2	< 0.001	5.2
Storage Type	1	< 0.001	5.6	1	< 0.001	0.9	1	0.429	0.1	< 0.001	23.2	< 0.001	2.2	0.010	1.2
Storage Duration	3	0.010	0.3	3	< 0.001	72.6	3	0.001	2.4	0.003	1.1	< 0.001	20.0	< 0.001	10.5
Site × Year	6	< 0.001	5.2	6	< 0.001	0.6	–	–	–	–	–	–	–	–	0.0
Site × Crop Type	6	< 0.001	1.8	6	< 0.001	1.3	–	–	–	–	–	–	–	–	0.0
Site × Genotype	22	< 0.001	1.0	22	< 0.001	0.6	–	–	–	–	–	–	–	–	0.0
Site × St. Type	6	< 0.001	9.1	6	< 0.001	0.7	–	–	–	–	–	–	–	–	0.0
Site × St. Duration	18	< 0.001	9.7	18	< 0.001	2.6	–	–	–	–	–	–	–	–	0.0
Year × Crop Type	1	< 0.001	0.5	1	< 0.001	0.1	1	0.139	0.3	0.001	1.2	< 0.001	2.4	< 0.001	13.9
Year × Genotype	6	< 0.001	0.2	6	< 0.001	0.2	4	< 0.001	7.2	< 0.001	13.0	< 0.001	6.9	0.007	2.6
Year × St. Type	1	< 0.001	0.9	1	0.005	0.0	1	< 0.001	5.2	0.775	0.0	0.002	1.0	0.009	1.2
Year × St. Duration	3	< 0.001	0.3	3	< 0.001	0.2	3	0.674	0.2	0.236	0.3	0.105	0.6	0.975	0.0
Crop Type × Genotype	–	–	–	–	–	–	–	–	–	–	–	–	–	–	0.0
Crop Type × St. Type	1	< 0.001	0.2	1	0.857	0.0	1	0.385	0.1	0.004	0.7	< 0.001	15.3	< 0.001	5.0
Crop Type × St. Duration	3	0.943	0.1	3	0.013	0.1	4	0.049	1.3	0.979	0.0	< 0.001	2.8	< 0.001	4.0
Genotype × St. Type	6	0.589	0.1	6	0.017	0.0	3	< 0.001	4.6	< 0.001	1.9	0.003	1.5	0.273	0.7
Genotype × St. Duration	18	0.646	0.5	18	< 0.001	0.4	12	< 0.001	9.6	0.421	1.0	0.024	2.5	0.012	4.7
St. Type × St. Duration	3	< 0.001	1.3	3	< 0.001	0.3	3	0.019	1.3	< 0.001	3.4	0.943	–	0.338	0.6
Variance explained (%)			*61.8*			*87.8*			58.5		77.9		*70.9*		*58.0*

*Model and error degrees of freedom were118 and 2,569 for moisture content and biomass loss, and 42 and 236 for composition analysis. Data were arcsine-transformed for ANOVA.

### Biomass moisture dynamics under aerobic storage

**Fig. 2:**
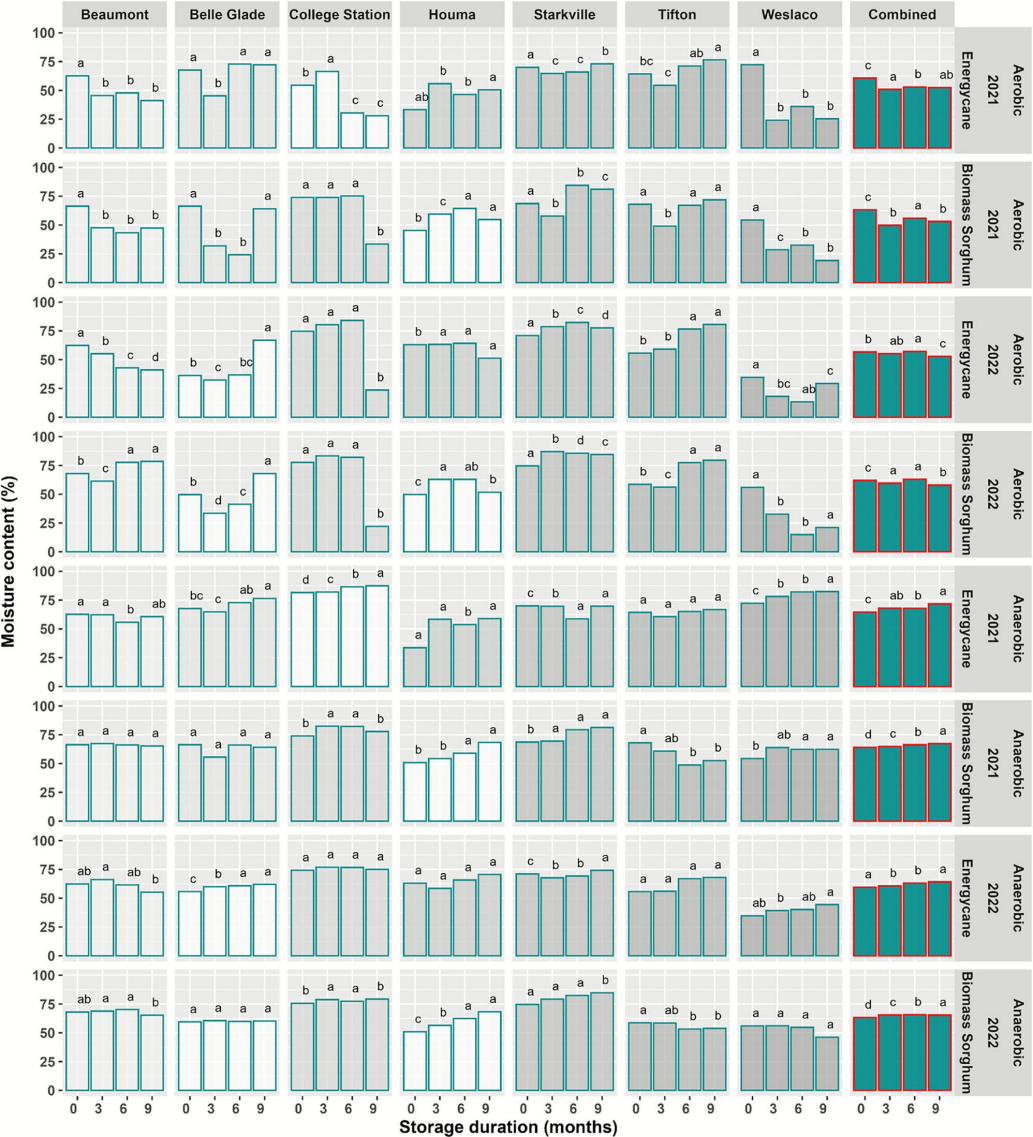
Change in moisture content during aerobic and anaerobic storage of machine-harvested energycane and biomass sorghum across seven experiment sites (Beaumont-TX, Belle Glade-FL, College Station-TX, Houma-LA, Starkville-MS, Tifton-GA, Weslaco-TX). Color bars for individual site and year combinations indicate entries based on observed data. White bars are based on estimation using missMDA (Josse and Husson^28^). Moisture contents for different storage durations in each subplot having the same lowercase letter are not significantly different from each other at 0.05 with Tukey’s HSD multiple comparison test.

Under aerobic storage, there was considerable variability in biomass moisture dynamics across different sites and years. For a specific site and year combination, moisture dynamics showed a similar pattern between energycane and biomass sorghum (Fig. 2).

In 2021, biomass moisture decreased during storage for Beaumont, College Station, and Weslaco. But for Belle Glade, Starkville, and Tifton, biomass moisture dropped 3 months post storage and increased again 6 and 9 months post storage. Biomass moisture for Houma increased during storage, probably due to the wet weather condition during storage^29^. Averaged over all seven sites, there was a significant decrease in biomass moisture 3 months post storage for both energycane and biomass sorghum, with moisture slightly increasing 6 months post storage and then dropping slightly 9 months post storage (Fig. 2). Average moistures for energycane were 61.2, 51.0, 53.1 and 52.4% after 0, 3, 6 and 9 months of storage, respectively. Corresponding average moistures for biomass sorghum were 63.4, 49.7, 56.1 and 53.6%.

In 2022, biomass moisture for energycane in Beaumont decreased during storage, but moisture for biomass sorghum decreased 3 months post storage and increased 6 and 9 months post storage (Fig. 2). For all other six sites, moisture showed a similar pattern between energycane and biomass sorghum but varied between sites. For Belle Glade, Tifton and Weslaco, biomass moisture dropped 3 months post storage but increased again 6 and 9 months post storage. For College Station, Houma and Starkville, biomass moisture increased from 3 to 6 months post storage and then dropped 9 months post storage. The overall moisture pattern in 2022 was similar to that in 2021. There was a significant decrease in biomass moisture 3 months post storage for both energycane and biomass sorghum, and moisture had a slight increase 6 months post storage and dropped slightly 9 months post storage (Fig. 2). Average moistures for energycane were 56.8, 55.3, 57.2 and 52.9% after 0, 3, 6 and 9 months of storage, respectively. Corresponding average moistures for biomass sorghum were 62.1, 59.6, 63.2 and 58.0%.

### Biomass moisture dynamics under anaerobic storage

The moisture content under anaerobic storage showed much less variability across sites and years, with energycane and biomass sorghum showing very similar patterns (Fig. 2). For most site-year combinations, moisture increased slightly through storage. Noticeable exceptions include Tifton for biomass sorghum in 2021, which showed a significant drop for 6 and 9 months post storage probably due to anaerobic storage bags being compromised. Averaged over all seven sites, moistures for energycane were 64.6, 68.0, 67.8 and 71.8% after 0, 3, 6 and 9 months of storage in 2021 and 59.5, 60.6, 63.0 and 64.2% in 2022, respectively. Corresponding average moistures for biomass sorghum were 64.1, 64.9, 66.3 and 67.4% in 2021 and 63.3, 65.5, 65.8 and 65.4% in 2022. Moisture increased significantly through storage except for biomass sorghum in 2022, which showed a 0.4% drop from 6 to 9 months post storage, probably due to the large moisture drop in Weslaco (Fig. 2). When data are pooled across years and crops, moisture increased significantly from 62.9 to 67.2% over the 9 months of storage. The increased moisture is probably due to accumulation of metabolic water^30^. The biomass in silage bags was not tightly packed, leaving air pockets in the biomass material. Plant cells continue to respire, consuming oxygen from the air pockets and producing water and carbon dioxide, leading to an increase in moisture within the silage bags.

### Analysis of variance on biomass loss during storage

Each main effect factor had a significant impact on biomass loss, with storage duration, site, and storage type accounting for 72.6, 5.6 and 0.9% of the total variability, respectively (Table 1). Crop type had the least impact, accounting for only 0.1% of the variability, suggesting similar biomass loss pattern between energycane and biomass sorghum. The top three two-way interactions that affect biomass loss were site × storage duration, site × crop type, and site × storage type, accounting for 2.6, 1.3 and 0.7% of the total variability, respectively (Table 1). The main effects and two-way interactions accounted for 87.8% of the total variability (Table 1). All main effects and two- and three-way interactions accounted for 92.2% of the total variability.

### Dry biomass loss under aerobic and anaerobic storage

**Fig. 3:**
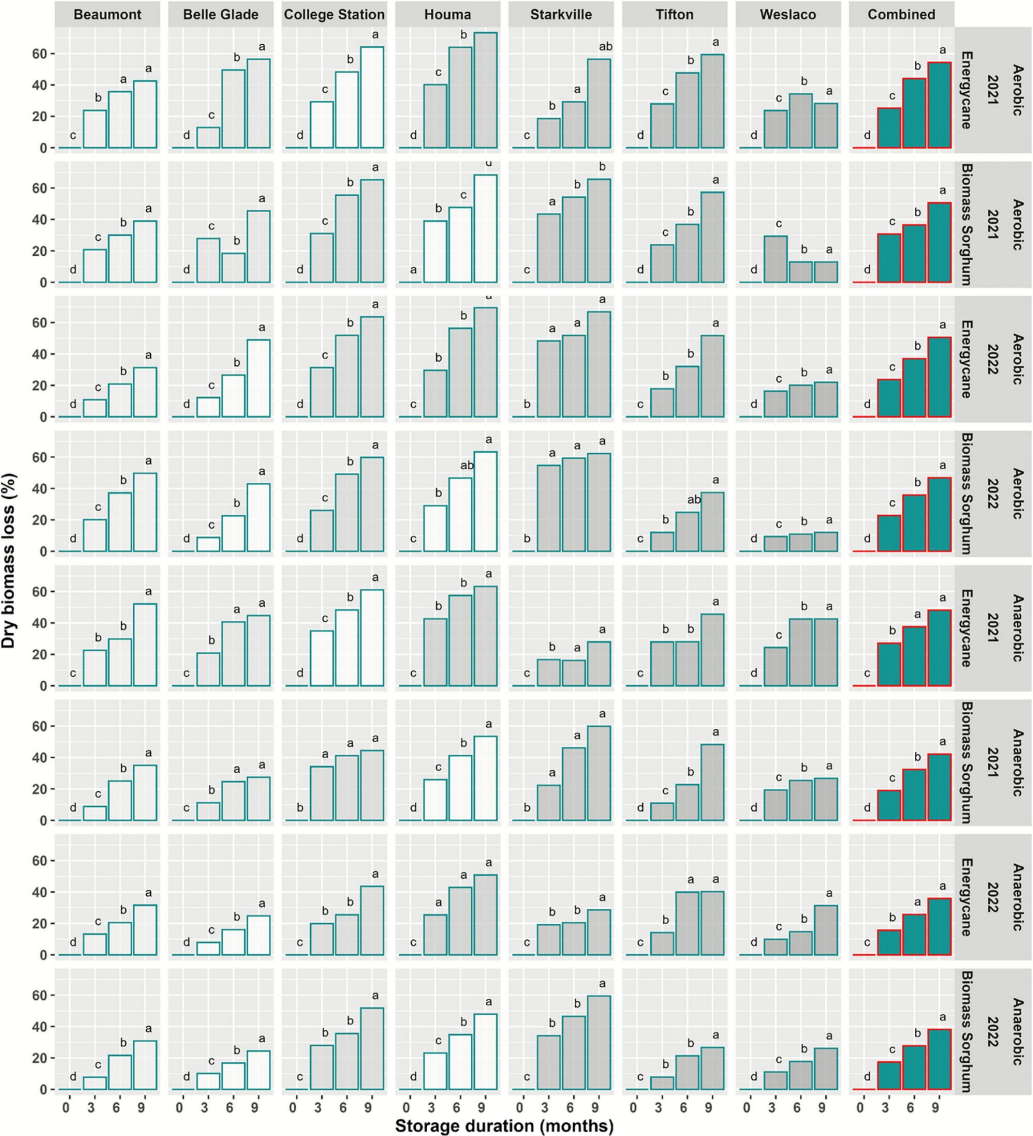
Biomass loss during aerobic and anaerobic storage of machine-harvested energycane and biomass sorghum across seven experiment sites (Beaumont-TX, Belle Glade-FL, College Station-TX, Houma-LA, Starkville-MS, Tifton-GA, Weslaco-TX). Color bars for individual site and year combinations indicate entries based on observed data. White bars are based on estimation using missMDA (Josse and Husson^28^). Biomass losses for different storage durations in each subplot having the same lowercase letter are not significantly different from each other at 0.05 with Tukey’s HSD multiple comparison test.

Biomass loss increased with storage duration (Fig. 3). This general pattern was consistent across sites and years for both energycane and biomass sorghum and for both aerobic and anaerobic storage.

Averaged across all sites, biomass losses after 3, 6 and 9 months of aerobic storage were 25.2, 44.1 and 54.5% in 2021 and 23.4, 35.7 and 50.0% in 2022 for energycane, respectively. Corresponding values were 30.9, 36.4 and 50.7% in 2021 and 23.3, 34.6 and 44.6% in 2022 for biomass sorghum (Fig. 3).

Averaged across sites, biomass losses after 3, 6 and 9 months of anaerobic storage were 26.9, 37.2 and 47.7% in 2021 and 15.2, 23.5 and 33.7% in 2022 for energycane, respectively. Corresponding values were 19.6, 33.2 and 42.6% in 2021 and 19.2, 26.5 and 37.0% in 2022 for biomass sorghum (Fig. 3).

**Fig. 4:**
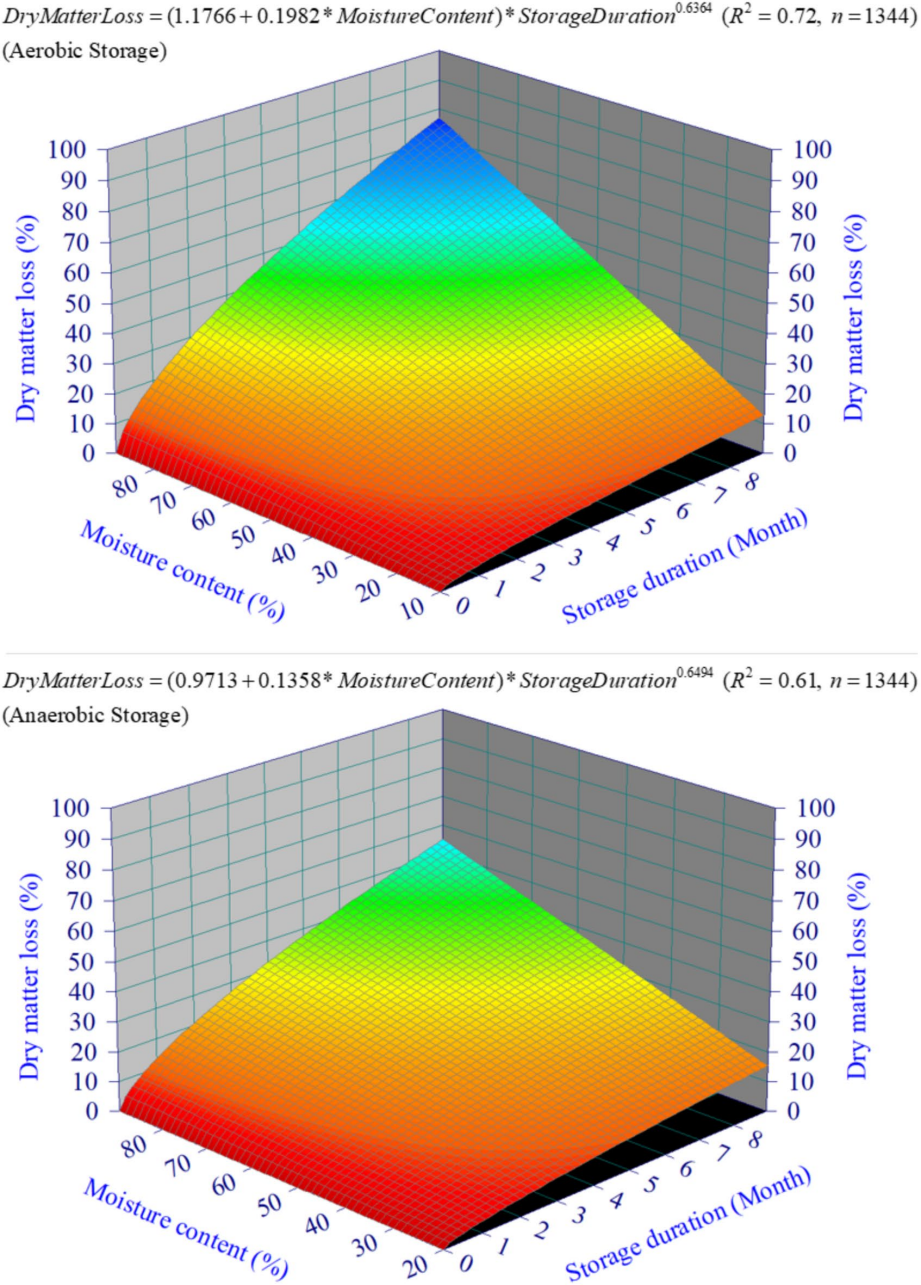
Response surface of dry matter loss as a power function of storage moisture and storage duration under aerobic and anaerobic storage.

Averaged across all sites, years and crop types, biomass losses after 3, 6 and 9 months of aerobic storage were 25.7, 37.7 and 49.9%, respectively. Corresponding values were 20.2, 30.1 and 40.3% under anaerobic storage (Fig. 3). Biomass loss as a power function of storage duration and moisture content is shown in Fig. 4. The power terms $StorageDuration^{0.6364}$ for aerobic storage and $StorageDuration^{0.6494}$ for anaerobic storage indicate decreasing rate of loss over storage duration. The linear terms $0.1982*MoistureContent$ for aerobic storage and $0.1358*MoistureContent$ for anaerobic storage indicate a linear rate of loss with increasing moisture content. The response surface shows a much greater dry matter loss towards higher moisture.

### Analysis of variance on composition change during storage

Except for year and storage type on cellulose, all main effects of year, crop type, genotype, storage type and storge duration had significant impact on cellulose, hemicellulose, lignin, and ash (Table 1).

For cellulose, the top 3 main effect factors were crop type (24.8%), storage duration (2.4%), and genotype (1.5%) (Table 1). The top three two-way interactions were genotype × storage duration (9.6%), year × genotype (7.2%), and year × storage type (5.2%). The main effects and two-way interactions accounted for 58.5% of the total variability. All main effects and two- and three-way interactions accounted for 74.7% of the total variability.

For hemicellulose, the top 3 main effects were crop type (25.7%), storage type (23.2%), and year (4.9%) (Table 1). The top three two-way interactions were year × genotype (13.0%), storage type × storage duration (3.4%), and genotype × storage type (1.9%). The main effects and two-way interactions accounted for 77.9% of the total variability. All main effects and two- and three-way interactions accounted for 88.0% of the total variability.

For lignin, the top three main effects were storage duration (20.0%), crop type (12.7%), and genotype (2.2%) and storage type (2.2%) (Table 1). The top three two-way interactions were crop type × storage type (15.3%), year × genotype (6.9%), and crop type × storage duration (2.8%). The main effects and two-way interactions accounted for 70.9% of the total variability. All main effects, and two- and three-way interactions accounted for 85.6% of the total variability.

The top three main effects for ash were storage duration (10.5%), year (7.4%), and genotype (5.2%) (Table 1). The top three two-way interactions were year × crop type (13.9%), crop type × storage type (5.0%), and genotype × storage duration (4.7%). The main effects and two-way interactions accounted for 58.0% of the total variability. All main effects, two, and three-way interactions accounted for 76.9% of the total variability.

### Composition change during aerobic and anaerobic storage

**Fig. 5:**
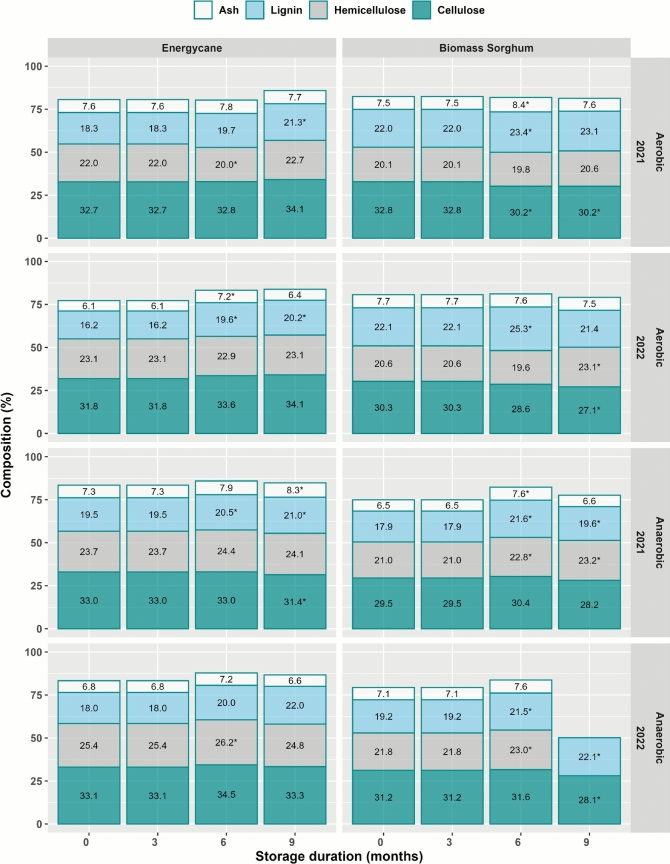
Biomass composition changes under aerobic and anaerobic storage. Composition values followed by ^*^ are significantly different from those at 0 month.

Biomass composition for cellulose, hemicellulose, lignin, and ash changed little over the 9-month storage (Fig. 5). Averaged across years and crop types, cellulose varied from 31.4 to 31.9% during 9 months of aerobic storage, hemicellulose varied from 20.8 to 22.3%, lignin varied from 19.6 to 21.4%, and ash varied from 7.1 to 7.7%. Under anaerobic storage, cellulose varied from 30.4 to 32.2%, hemicellulose varied from 23.0 to 24.0%, lignin varied from 18.7 to 21.1%, and ash varied from 6.9 to 7.7% (Fig. 5). Average change in any composition attribute was less than 3%. There were significant differences in composition between energycane and biomass sorghum. Average cellulose were 30.3% and 33.0% for energycane and biomass sorghum, respectively. Corresponding values were 21.3% and 23.4% for hemicellulose, 19.2% and 21.0% for lignin, and 7.1% and 7.4% for ash.

**Fig. 6:**
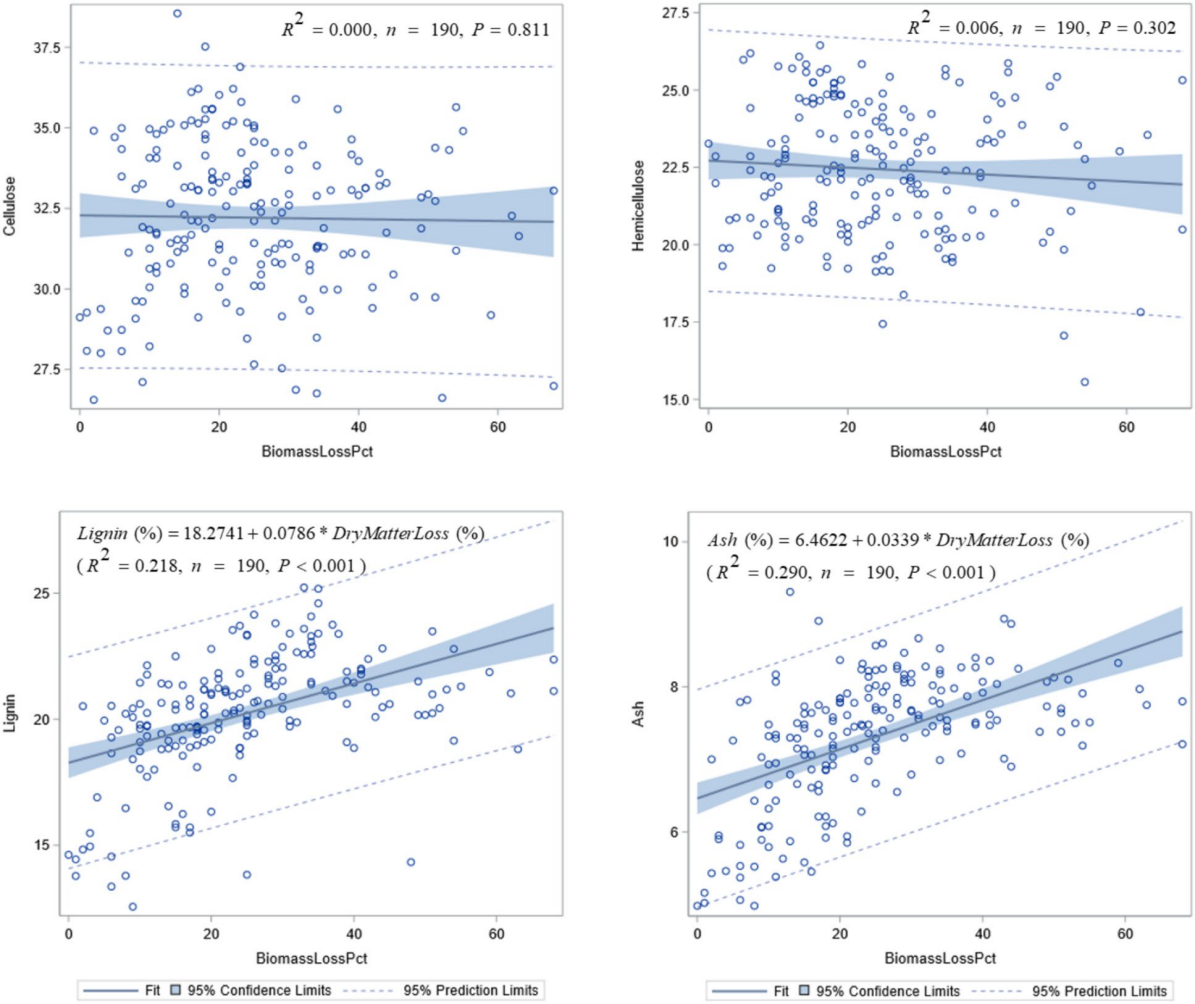
Relationship between biomass composition and dry matter loss during storage. Regression equations for cellulose and hemicellulose are omitted due to insignificant correlations.

There was no significant correlation between biomass loss and cellulose ($R^{2} = 0.000,P = 0.811$ ) nor hemicellulose ($R^{2} = 0.006,P = 0.302$) (Fig. 6). But there was a significant correlation between biomass loss and lignin ($R^{2} = 0.218,P < 0.001$) or ash ($R^{2} = 0.290,P < 0.001$); increasing biomass loss was associated with increasing lignin and ash contents (Fig. 6).

### Impact of environmental factors on biomass characteristics during storage

**Fig. 7:**
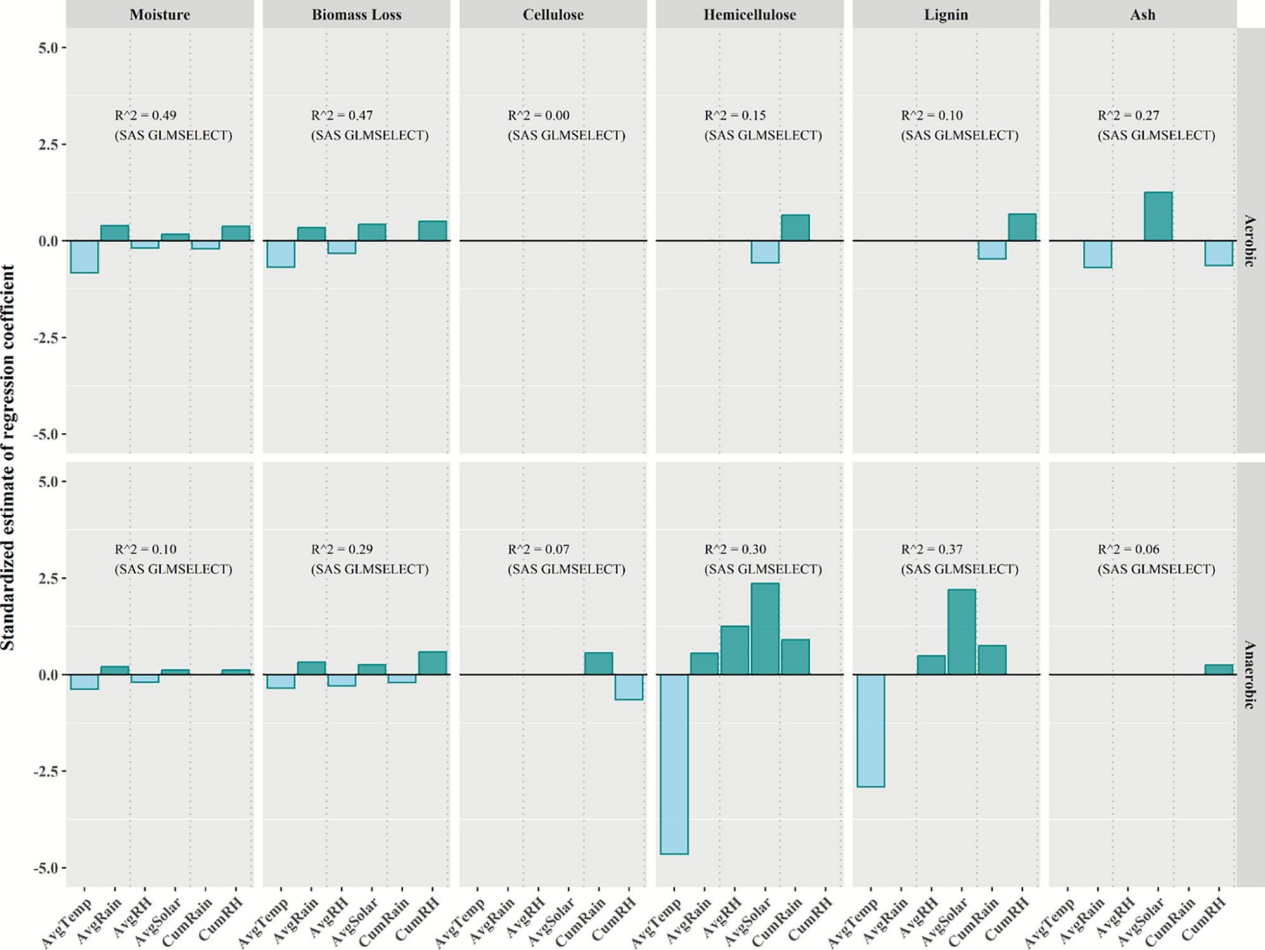
Impacts of environmental covariates on biomass characteristics during aerobic and anaerobic storage based on SAS GLMSELECT stepwise regression. Darker color bars represent positive effects and lighter color bars represent negative effects.

Site and storage duration had the greatest impact on biomass storage characteristics. For both aerobic and anaerobic storage, moisture content and biomass loss were positively correlated with average rainfall, average solar radiation, and cumulative relative humidity, and negatively correlated with average temperature and average relative humidity (Fig. 7). There was considerable variability in the impact of environmental covariates on composition attributes. There was no environmental covariate that had a consistent positive or negative impact on all composition attributes for both aerobic and anaerobic storage.

Except for moisture content and biomass loss under aerobic storage, which had relatively large *R*^2^ values (0.49 for moisture and 0.47 for biomass loss), *R*^*2*^ values for composition attributes (cellulose, hemicellulose, lignin, and ash) were all very low (Fig. 7). The relatively low regression *R*^2^ values suggest limited impact of environmental covariates on biomass storage characteristics, probably due to some degree of insulation from the storage piles and bale bags.

## Discussion

### Biomass loss under aerobic storage

Research on dry matter loss of herbaceous cellulosic crops during storage has mostly focused on corn stover^12,19–21^, switchgrass^22–25^, and forage or sweet sorghum^27^. Shinners et al.^20^ reported 3.3% dry matter loss in covered piles of chopped corn stover over a duration of 238 days. The low biomass loss was attributed to low initial moisture of 22.9%, but dry matter loss in uncovered piles was 39.1% due to high precipitation infiltration^20^.

In a study on switchgrass involving two bale shapes (round and square bale), three storage surfaces (gravel pad, wooden pallet, and grass sod), and two cover types (covered and uncovered), Mooney et al.^15^ reported storage losses ranging from 9.8 to 41.9% with an average storage duration of 300 days, but no information on bale moisture was provided. Coble and Egg^31^ reported 18% dry matter loss for outdoor storage of sweet sorghum bales with an average of 20.5% moisture over 165 days of storage.

In our study, dry matter loss under aerobic storage piles was 49.9% after 270 days of storage. The loss is higher than reported using bales for corn stove, switchgrass, and sweet sorghum with or without covers. The higher dry matter loss was probably due to higher moisture content of biomass during storage (59.9% for energycane and 58.4% for biomass sorghum) (Fig. 2). In a study using bunker silos for corn silage with over 80% moisture content, Ashbell and Weinberg^32^ reported greater than 60% biomass loss after 8 months of storage, which is comparable to the results observed in our study. Wendt et al.^19^ similarly reported high dry matter loss of approximately 30% over a 111-day aerobic storage period in laboratory reactors.

**Fig. 8:**
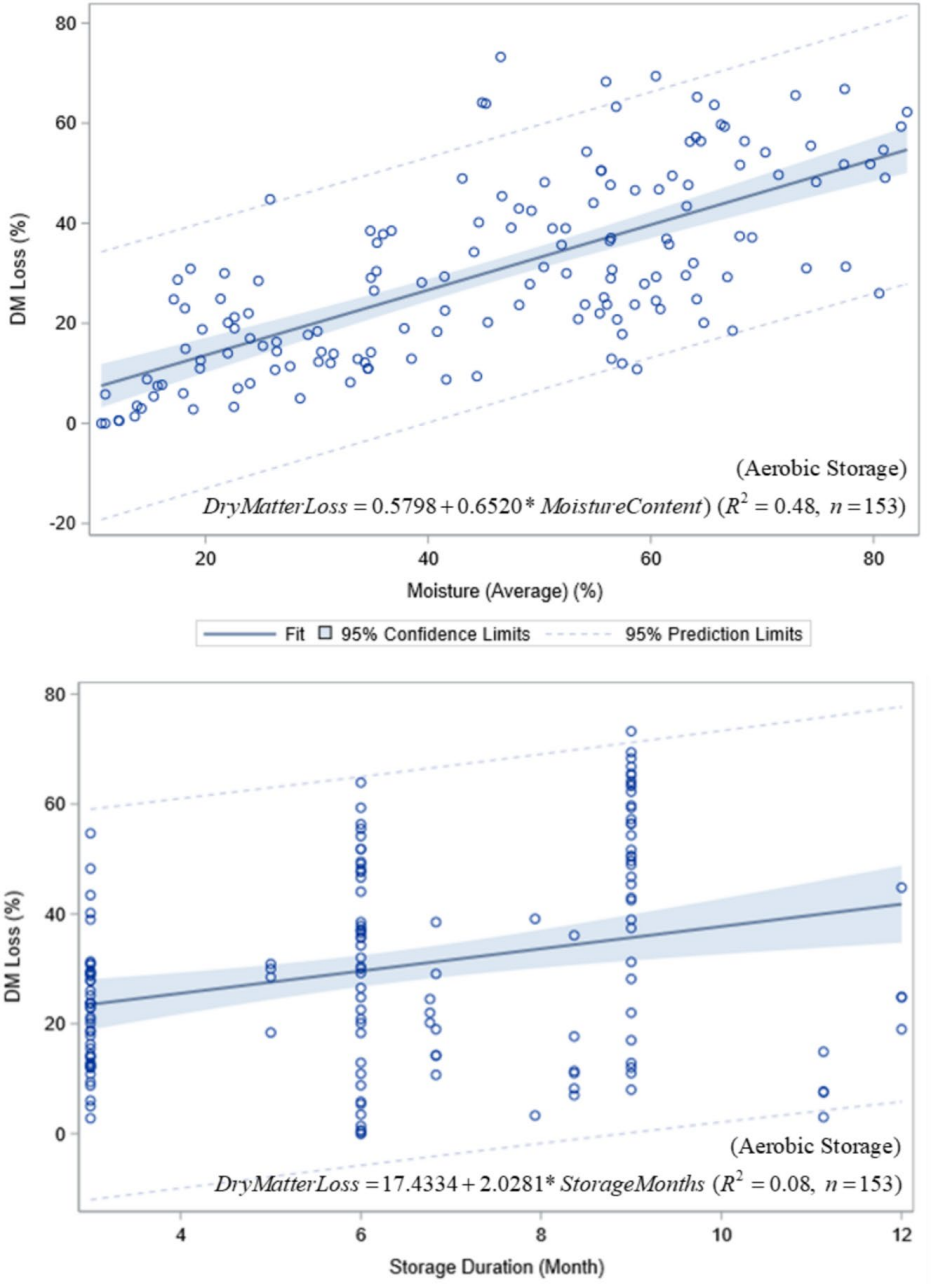
Dry matter loss vs moisture content (top plot) and storage duration (bottom plot) under aerobic storage (Data source: this study, Sinners et al. 2007, 2010, 2011, Shah et al. 2011, Khanchi et al. 2013, and Smith et al. 2013).

Combining data from this study and results from similar studies under aerobic conditions, we found a strong relationship between biomass loss and average moisture content during storage, with higher moisture content leading to greater dry matter loss (Fig. 8). The relationship between dry matter loss and storage duration was significant but much weaker (Fig. 8). Higher dry matter loss with higher moisture emphasizes the importance of moisture control as a strategy to limit biological degradation in aerobic storage. Moisture distribution in storage is not uniform in time or location but varies depending on the storage conditions. Precipitation and infiltration can add moisture to the storage system leading to changes in the bulk moisture content and dry matter loss^12^.

### Biomass loss under anaerobic storage

Shinners et al.^20^ evaluated dry matter losses of chopped corn stover under anaerobic storage using sealed silo bags. The authors reported dry matter loss of 0.2–0.9% over a duration of over 200 days. The authors also reported that losses in silo bags were similar to those under aerobic treatments when anaerobic conditions were not maintained in the bags.

In a farm-scale anaerobic storage study using field dried and chopped switchgrass and reed canarygrass in sealed silo bags, Williams and Shinners^22^ found an average storage loss of 2.7 and 2.2% for switchgrass and reed canarygrass, respectively, over a storage duration of 220 days. The moisture content into storage was 54.1% for switchgrass and 43.4% for reed canarygrass. Shinners et al.^20^ reported less than 5% dry matter loss in a series of experiments on corn stover stored 7–9 months in anaerobic silo bags across a wide range of moisture and ambient conditions. The pH values of their storage materials were mostly less than 5.0.

Through analysis on the relationship between storage moisture and pH values, Shinners et al.^20^ suggested that if the goal is to achieve a pH of 4.0–4.5, the desired harvest and storage moisture would be in the range of 35–45% for anaerobic storage of cellulosic materials. The combination of anaerobic conditions and the presence of organic acids and corresponding low pH serve to reduce the overall microbial activity in ensiled systems^11^. Sufficient fermentable soluble sugars present at the time of ensiling is necessary to support organic acid production and pH reduction^11^.

In our study, dry matter losses after 9 months of storage were approaching 40%, which is much greater than those reported in ensiled silo bags. The pH values of the storage material at the Beaumont site increased from 5.8 to 6.8 under aerobic storage and increased from 5.3 to 6.9 under anaerobic storage, suggesting that the anaerobic condition was not maintained through the storage duration, probably due to: (1) the relatively small volume of the material, (2) relatively large air space in the unpacked storage material with high porosity and high oxygen content^33^, and (3) compromised silage bags due to wind and other inclement storage conditions. Since similar dry matter loss patterns were observed for anaerobic storge trials across all seven sites, anaerobic storage conditions probably also apply to the other sites. Our results from the bale bags most probably reflect the biomass characteristics under semi-anaerobic storage conditions. For commercial-scale biorefinery operations, large-scale biomass storage systems will be needed to support year-round biomass supply. A major challenge will be to design storage systems that can minimize biomass loss by maintaining anaerobic conditions and minimizing aerobic exposures. Greater biomass losses will require higher storage capacity and larger feedstock production footprint. A detailed regional analysis on how storage conditions impact biomass loss and feedstock supply is warranted but beyond the scope of this study.

### Dry matter loss pattern vs storage duration and moisture content

Most previous studies have assessed dry biomass loss for a single storage period^20–22,25,27,31,34^. Several studies have examined the relationship between dry matter loss and storage duration^12,14,15,23,26,35^. Sanderson et al.^23^ found that dry matter loss of baled switchgrass increased linearly with time in storage. The baled switchgrass was field-dried to 11–19% moisture and there was no heating due to microbial activity. In a study using three particle sizes of switchgrass and two types of bale wraps, Yu et al.^26^ found that dry matter losses of switchgrass bales also increased linearly with days in storage. The moisture content of the bales into storage was ~ 22%. Mooney et al.^15^ reported that dry matter losses for baled switchgrass increased at a diminishing rate and approached an asymptotic maximum over time. The authors found that non-linear quadratic plateau and Mitscherlich-Baule models appear superior to the linear model in mimicking biomass loss over time. Shah et al.^14^ also reported that most of the dry matter loss for baled corn stover occurred early during the storage. A similar trend was observed in a mini-silo storage study of switchgrass^35^ and in a study on covered open-air storage piles of fine and coarse wood chips^36^. The pattern of rapid early and slower late biomass loss was attributed to the early consumption of easily accessible carbohydrates by microorganisms, leaving behind the resistant compounds such as cellulose, hemicellulose and lignin, leading to a slower process of degradation^37^.

In this study, a power-response function with decreasing rate of biomass loss provided a better fit to the dry matter loss over time than a linear response function (Fig. 4). The linear to non-linear response of dry matter loss over storage duration might be partially attributed to the high moisture content of the storage materials, with a more pronounced non-linear pattern at higher moisture contents (~ 60% in this study and in^36^, and ~ 63% in^35^). With low moisture content in storage materials, activities of plant enzymes and aerobic microorganisms are usually greatly reduced, resulting in minimal internal heating and consumption of accessible carbons, and thus a linear response to storage time. Increased enzymatic and microorganism activities at higher moisture contents may lead to a non-linear response to storage time. But further studies are needed to quantify the response curves in terms of storage methods and storage conditions.

### Biomass composition change during storage

This study only quantified the biomass, moisture, and composition changes during storage but did not explore the underlying causes for these changes. Further studies would be to quantify the microbial community and microbial degradation processes as impacted by biomass quality and environmental factors. Results from such studies will facilitate the development of mechanistic models that can predict biomass loss and composition changes under different environmental conditions. Microbial degradation of aerobically stored biomass materials can be characterized by CO_2_ production, microbial heat generation and the resulting temperature increase, and dry matter loss^11,17,38,39^. Aerobic microbial degradation by bacteria, yeast, and fungi consumes easily-digestible carbohydrates, leaving behind material enriched in non-fermentable biomass components^11^, following the general path of digestion: soluble carbohydrates → structural carbohydrates (cellulose and hemicellulose) → lignin^40^. Lignin content often proportionally increases as a result of storage loss due to the biodegradation of cellulose and hemicellulose and the inaccessibility of lignin to microorganisms^19,20^. Ash content may also increase due to the loss of organic components through biological degradation by microorganisms and chemical processes, leaving behind the inorganic components, resulting in a higher ash content^41^.

In evaluating dry matter losses of chopped corn stover under covered aerobic storage piles over a duration of 238 days, Shinners et al.^20^ reported 3.3% dry matter loss, 4.5% increase in cellulose, 1.9% decrease in hemicellulose, 0.9% increase in lignin, and 4.2% decrease in ash content. The authors also reported that for most constituents, there was significantly less change for anaerobically stored corn stover. In a storage study on corn stover bales, Shah et al.^14^ reported 4% increase in cellulose, − 2 to 1% change in hemicellulose, and 3% increase in lignin. Shinners et al.^25^ reported ~ 2% increase in cellulose and ~ 1% increase in hemicellulose after 293 days of outdoor storage of switchgrass and reed canarygrass bales. Similar results were reported for aerobically stored corn stover^16,19,42^. Increase in lignin is particularly disadvantageous in cellulosic-ethanol biorefineries that do not utilize the lignin fraction during the conversion process^13^.

Similar trends in composition changes were observed in our study for both aerobic and anaerobic storage. Since the observed biomass losses were much greater than the above-mentioned studies, it was not clear why we did not observe a much greater change in composition characteristics. If the magnitude of composition change remains small through extended storage, supplying the stored materials to biorefineries will likely require little change in the down-stream conversion processes, with minimal impact on the quality and quantity of fuel production. In a study on energycane, Knoll et al.^10^ reported that the composition of biomass fiber (cellulose, hemicellulose, and lignin) from just-in-time harvested biomass was generally stable over time post maturity, and high-fiber energycane can be harvested later during the winter months with little change in conversion properties.

### Dry vs wet storage

Wet storage systems are based on chopping herbaceous biomass in the field at moisture contents between 40 and 65% (wet basis) and storing the biomass in silage bags, bunkers, silos, or drive-over piles to limit access to oxygen and preserve biomass^11^. Wet anaerobic storage systems are an alternative to dry storage and have consistently and successfully demonstrated biomass preservation in long term storage for livestock feed and forage^11^. Ensiled biomass remains stable for months to years if anaerobic conditions are maintained^11^. Expected dry matter losses under best management practices range from 6 to 15% depending on storage structure, with losses as low as 3%^11,33^.

Silage production usually involves a field dry-down (or wilting) phase to reduce moisture and enhance ensiling characteristics. Traditional hay making is restricted to crops that can dry quickly and uniformly, and to areas with little or no rainfall during harvest^18^. Crop residues, such as corn stover for bioenergy production, are available only at the time of grain harvest and accordingly contains lower initial moisture contents and lower soluble sugars compared to feedstock dedicated for forage^43,44^. In addition, feedstock for forage requires the preservation of leaf materials due to its higher nutritional values, but for wet storage of cellulosic biomass, it may be best to return the majority of the leaf materials to the fields to recover the nutrients in leaves. This may impact the wet storage options.

For crop residues, moisture content at the time of harvest can be as high as 75% to less than 20% depending on the geographic locations (climate conditions) and stage of harvest^45^. A spatial analysis by Oyedeji et al.^45^ using hourly weather data concluded that 37.2% of corn stover has less than 20% moisture content, 27.0% has moisture content between 20 and 40%, and 36.5% has greater than 40% moisture content nationwide. Average moisture into storage in our study was ~ 60% for energycane and biomass sorghum. Field drying of energycane and biomass sorghum is costly due to high yields, weather risks, energy inputs, and harvest timeliness^22^. Wet storage using silage bags, bunkers, silos, or drive-over piles will likely the most feasible storage option to address the year-round biomass supply challenge for biorefinery development in the southeastern US.

## Conclusion

This paper presents the results of a study on biomass characteristics of energycane and biomass sorghum under aerobic and anaerobic storage. Crop type and genotype accounted for less than 3% of the observed variability in moisture content and dry matter loss, suggesting similar storage characteristics between energycane and biomass sorghum. Dry matter loss was mostly impacted by storage duration, while moisture content was mostly impacted by site. Biomass moisture under covered aerobic storage piles tended to decline but with considerable variability across sites and seasons. Biomass moisture under anaerobic silage bags showed a small but consistent increase during storage. Average moisture content decreased from 60.7 to 50.4% after 9 months of aerobic storage but increased from 62.9 to 67.2% for anaerobic storage. Dry matter loss averaged 49.9% after 9 months of aerobic storage and 40.3% for anaerobic storage. Dry matter loss over storage duration and moisture can be described as a non-linear power function of storage duration and a linear response term over average moisture during storage. Average changes in biomass composition for cellulose, hemicellulose, lignin, and ash were all less than 3% during the 9-month storage regardless of storage types. This paper represents the first comprehensive study on storage characteristics of energycane and biomass sorghum. It provides valuable insights on changes in feedstock quantity and quality during storage and fills a critical knowledge gap in addressing the challenge of year-round biomass supply for biorefinery development.

## Materials and methods

### Site descriptions

Storage trials were conducted in seven sites for energycane and six sites for biomass sorghum across five states in the southeastern US, including Texas (Beaumont, College Station, and Weslaco), Mississippi (Starkville), Georgia (Tifton), Florida (Belle Glade), and Louisiana (Houma: energycane only) (Table 2). The latitude ranges from 26.2° N (Weslaco) to 33.4° (Starkville), and longitude ranges from 80.6° W (Belle Glade) to 97.9° W (Weslaco). Annual precipitation in 2021–2023 ranged from 456 mm in Weslaco to 1,167 mm in Beaumont. These sites are representative of the range of environmental and production conditions in the southeastern US.

**Table 2:** Average weather conditions during the storage seasons from September 1, 2021 to August 31, 2023 across the seven experiment sites.

Site	State	Lat (°N)	Long (°W)	Elevation (m)	Annual precipitation (mm)	Daily average		
						Temperature (°C)	Humidity (%)	Radiation (MJ/m^2^)
Weslaco	Texas	26.2	97.9	20	456	24.3	66.1	16.1
Belle Glade	Florida	26.7	80.6	3	1,077	23.7	67.0	17.2
Houma	Louisiana	29.6	90.8	1	1,149	21.3	66.5	16.5
Beaumont	Texas	30.1	94.3	10	1,167	21.5	77.3	16.8
College Station	Texas	30.5	96.4	67	854	22.3	64.2	16.8
Tifton	Georgia	31.5	83.6	107	669	20.1	70.2	15.2
Starkville	Mississippi	33.4	88.7	79	817	18.4	70.5	16.1

### Energycane and biomass sorghum genotypes

Six of the seven sites grew energycane genotypes Ho 01-08, Ho 02-113, and Ho 06-9002. Due to quarantine restriction, Belle Glade grew Ho 02-113, UFCP 84-1047, and UFCP 87-0053. The first three genotypes were developed by the USDA-ARS Sugarcane Research Unit in Houma, Louisiana^46^, while the other two were jointly developed by the USDA-ARS Sugarcane Field Station at Canal Point in Florida and the University of Florida^47,48^. Three photoperiod-sensitive hybrid genotypes of biomass sorghum were grown in each of the six sites, including TAM08001, TAM08005, and TAM08010, which were developed by the Sorghum Breeding Program at Texas A&M AgriLife Research in College Station, Texas.

In the southeastern US, energycane reaches its maximum biomass between late October and late December, while biomass sorghum reaches its maximum biomass between early October and early November, depending on locations.

Storage trials were initiated at the end of 2021 and 2022 crop seasons. Biomass was harvested with a forage chopper around crop maturity, with harvest dates ranging from late October to February the following year for energycane and from late September to mid-November for biomass sorghum. Due to issues with harvest equipment, biomass harvest in Weslaco was delayed to mid-February and early March of 2022 for the 2021 crop season for energycane and to early January and late February of 2023 for the 2022 crop season for biomass sorghum. Chopped biomass materials had a particle size of less than 1.0 cm except for some longer leaf segments and were immediately used for storage trials.

### Aerobic storage

Experimental designs for energycane and biomass sorghum production were described in^49,50^. The storage experiments were designed to evaluate the effect of aerobic and anaerobic storage on biomass quantity and quality for energycane and biomass sorghum. For each site, biomass from the recommended nitrogen treatment for a genotype was used for the storage experiment.

Chopped biomass was collected in a dump wagon during harvest and deposited in a pile on a 6.1 × 9.1 m^2^ woven poly tarp (0.127 mm thickness) covering the ground. The pile was then formed into a smooth contour using a tractor equipped with a front loader^51^. Each pile was then covered with another 6.1 × 9.1 m^2^ woven poly tarp, which was staked to the ground to prevent movement.

Each storage pile was approximately 2 m wide, 8 m long, and 1.5 m tall at the time of construction. One pile was built for each energycane and biomass sorghum genotype, for a total of six piles for each site (three piles for Houma which had only energycane). Four polypropylene mesh bags, each 56 × 46 cm^2^ with a square mesh of 3 × 3 mm, were filled with ~ 4 kg of chopped biomass, and inserted at three locations inside each pile at ~ 2 m apart and buried at approximately half the height of the pile. Mesh bags were sequentially removed from each pile after 3, 6 and 9 months of storage. Mesh bag removal started from one end of the pile and proceeded to the other end, which minimized disturbance to the bags that remained in the pile^51^.

### Anaerobic storage

Bale bags, each 1.2 m in diameter × 2.9 m long with 0.114 mm thickness, were filled with chopped biomass to a height of ~ 1.0 m. One bale bag was used for each storage duration and each energycane and biomass sorghum genotype, for a total of 18 bale bags for each site (2 crops × 3 genotypes × 3 storage durations). Houma had only energycane with 9 bale bags. A makeshift wooden frame was used to support the bale bag and a backhoe was used to load the chopped biomass into the bale bag. Four polypropylene mesh bags, as described above, filled with ~ 4 kg of chopped biomass, were inserted into the center of each bale bag during loading. The bale bags were then sealed with bale bag repair tape. The inserted mesh bags were collected by opening bale bags at 3, 6 and 9 months after storage.

### Biomass storage sample processing

Wet weights of the biomass samples at the time of storage were recorded and biomass samples were then dried at 65 °C to constant weight to estimate moisture content and dry matter. After storage, wet weight of each mesh bag was recorded immediately after collection and dried at 65˚C to constant weight to estimate moisture content. Biomass loss was calculated based on the estimated dry weight of the mesh samples before and after storage.

Oven-dried storage subsamples (~ 50 g) for each combination of crop, genotype, storage method, and storage duration were knife-milled using a Thomas Wiley® Cutting Mill (Model 4 Wiley® Mill; Thomas Scientific, Swedesboro, NJ). Composition analysis of the ground biomass samples (particle sizes < 500 µm) was conducted at the US Department of Energy’s National Renewable Energy Laboratory (NREL; Golden, CO) using Near-infrared (NIR) spectroscopy prediction models for lignocellulosic feedstocks^52^. For each sample, duplicate NIR composition predictions of lignin, glucan, xylan, arabinan, galactan, extractives, and ash were obtained using calibration models developed/optimized for herbaceous feedstocks^53–55^. The calibration models used partial least square (PLS) uncertainty analysis to identify/eliminate outlier predictions from abnormal samples. Biomass reference materials (including corn stover, switchgrass, miscanthus, sugarcane bagasse, and sorghum) whose composition profiles had previously been characterized/validated through interlaboratory comparisons^56^ were included among unknown samples to verify prediction accuracy.

### Data analysis

Analyses of variance for moisture content, biomass loss, and composition (cellulose, hemicellulose, lignin, and ash) were conducted using SAS 9.4 GLM^57^. Arcsine transformation^58^ was applied to all percentage data for analyses of variance. Main factors were site, year, crop, genotype, storage type, and storage duration. Treatment means were compared with Tukey’s multiple comparison test with α = 0.05^57^.

We calculated two sets of environmental covariates associated with site, year and storage duration: (1) mean weather factors during the storage duration and (2) cumulative weather factors during the storage duration. Weather factors included temperature, relative humidity, rainfall, and solar radiation, which were obtained from a weather station near each experiment site^59^. Collinearity between the environmental covariates was diagnosed using the COLLIN and VIF options in SAS REG^57^. Covariates with Condition Index exceeding 100 and VIF value exceeding 10 were excluded^60,61^. Thereafter, stepwise regression in SAS GLMSELECT^57^ was used to identify the most significant covariates impacting the storage characteristics, including AvgTemp (average temperature), AvgRain (average rainfall), AvgSolar (average solar radiation), AvgRH (average relative humidity), CumRain (Cumulative rain), and CumRH (Cumulative relative humidity). Dry matter loss (i.e. biomass loss) during storage was fitted to a power function $DML = (a + b*MoistureContent)*StorageDuration^{c}$, where $MoistureContent$ is average biomass moisture during storage on the wet basis, $StorageDuration$ is months in storage , and $a$, $b$, and *c* are parameters. Curve-fitting was conducted using TableCurve 3D V4 (SYSTAT Software Inc., Palo Alto, California, USA). Figures were prepared using either R^62^ or SAS^57^.

## Data Availability

Data will be made available on request (Contact author: Yubin Yang; [yyang@aesrg.tamu.edu](mailto:yyang@aesrg.tamu.edu)).

## References

[1] USDA. A USDA Regional Roadmap to Meeting the Biofuels Goals of the Renewable Fuels Standard by 2022 (https://www.usda.gov/sites/default/files/documents/USDA_Biofuels_Report_6232010.pdf) (Accessed 2/17/2025). (2010).

[2] Legendre, B. L. & Burner, D. M. Biomass production of sugarcane cultivars and early-generation hybrids. *Biomass Bioenerg.* **8**, 55–61. 10.1016/0961-9534(95)00014-x (1995).

[3] Fageria, N. K., Moreira, A., Moraes, L. A. C., Hale, A. L. & Viator, R. P. in Biofuel Crops: Production, Physiology and Genetics (ed Bharat P. Singh) 151–171 (CAB International, 2013).

[4] Rooney, W. L., Blumenthal, J., Bean, B. & Mullet, J. E. Designing sorghum as a dedicated bioenergy feedstock. *Biofuels Bioprod. Biorefin. –Biofpr.* **1**, 147–157. 10.1002/bbb.15 (2007).

[5] Lee, D. et al. Biomass production of herbaceous energy crops in the United States: Field trial results and yield potential maps from the multiyear regional feedstock partnership. *GCB Bioenergy* **10**, 698–716. 10.1111/gcbb.12493 (2018).

[6] Olson, S. N. et al. Energy sorghum hybrids: Functional dynamics of high nitrogen use efficiency. *Biomass Bioenerg.* **56**, 307–316. 10.1016/j.biombioe.2013.04.028 (2013).

[7] Olson, S. N. et al. High biomass yield energy sorghum: Developing a genetic model for C4 grass bioenergy crops. *Biofuels Bioprod. Biorefin. -Biofpr* **6**, 640–655. 10.1002/bbb.1357 (2012).

[8] Yang, Y. et al. Energycane growth dynamics and potential early harvest penalties along the Texas Gulf Coast. *Biomass Bioenerg.* **113**, 1–14 (2018).

[9] Dale, B. A sober view of the difficulties in scaling cellulosic biofuels. *Biofuels Bioprod. Biorefin. -Biofpr* **11**, 5–7. 10.1002/bbb.1745 (2017).

[10] Knoll, J. E. et al. Harvest date effects on biomass quality and ethanol yield of new energycane (Saccharum hyb.) genotypes in the Southeast USA. *Biomass Bioenergy* **56**, 147–156. 10.1016/j.biombioe.2013.04.018 (2013).

[11] Wendt, L. M. & Zhao, H. Y. Review on bioenergy storage systems for preserving and improving feedstock value. *Front Bioeng Biotechnol*10.3389/fbioe.2020.00370 (2020). 32411689 10.3389/fbioe.2020.00370 PMC7198811

[12] Smith, W. A., Bonner, I. J., Kenney, K. L. & Wendt, L. M. Practical considerations of moisture in baled biomass feedstocks. *Biofuels-Uk* **4**, 95–110. 10.4155/bfs.12.74 (2013).

[13] Darr, M. J. & Shah, A. Biomass storage: An update on industrial solutions for baled biomass feedstocks. *Biofuels-Uk* **3**, 321–332. 10.4155/bfs.12.23 (2012).

[14] Shah, A., Darr, M. J., Webster, K. & Hoffman, C. Outdoor storage characteristics of single-pass large square corn stover bales in Iowa. *Energies* **4**, 1687–1695. 10.3390/en4101687 (2011).

[15] Mooney, D. F., Larson, J. A., English, B. C. & Tyler, D. D. Effect of dry matter loss on profitability of outdoor storage of switchgrass. *Biomass Bioenerg.* **44**, 33–41. 10.1016/j.biombioe.2012.04.008 (2012).

[16] Khanchi, A., Jones, C. L., Sharma, B. & Huhnke, R. L. Characteristics and compositional change in round and square switchgrass bales stored in South Central Oklahoma. *Biomass Bioenerg.* **58**, 117–127. 10.1016/j.biombioe.2013.10.017 (2013).

[17] Wendt, L. M., Bonner, I. J., Hoover, A. N., Emerson, R. M. & Smith, W. A. Influence of airflow on laboratory storage of high moisture corn stover. *Bioenergy Research* **7**, 1212–1222. 10.1007/s12155-014-9455-3 (2014).

[18] Weinberg, Z. G. & Ashbell, G. Engineering aspects of ensiling. *Biochem. Eng. J.* **13**, 181–188. 10.1016/s1369-703x(02)00130-4 (2003).

[19] Wendt, L. M. et al. Compatibility of High-moisture storage for biochemical conversion of corn stover: Storage performance at laboratory and field scales. *Front Bioeng Biotechnol*10.3389/fbioe.2018.00030 (2018). 29632861 10.3389/fbioe.2018.00030 PMC5879930

[20] Shinners, K. J., Wepner, A. D., Muck, R. E. & Weimer, P. J. Aerobic and anaerobic storage of single-pass chopped corn stover. *Bioenergy Res.* **4**, 61–75. 10.1007/s12155-010-9101-7 (2011).

[21] Shinners, K. J., Binversie, B. N., Muck, R. E. & Weimer, P. J. Comparison of wet and dry corn stover harvest and storage. *Biomass Bioenerg.* **31**, 211–221. 10.1016/j.biombioe.2006.04.007 (2007).

[22] Williams, S. D. & Shinners, K. J. Farm-scale anaerobic storage and aerobic stability of high dry matter perennial grasses as biomass feedstocks. *Biomass Bioenerg.* **64**, 91–98. 10.1016/j.biombioe.2014.03.037 (2014).

[23] Sanderson, M. A., Egg, R. P. & Wiselogel, A. E. Biomass losses during harvest and storage of switchgrass. *Biomass Bioenerg.* **12**, 107–114. 10.1016/s0961-9534(96)00068-2 (1997).

[24] Monti, A., Fazio, S. & Venturi, G. The discrepancy between plot and field yields: Harvest and storage losses of switchgrass. *Biomass Bioenerg.* **33**, 841–847 (2009).

[25] Shinners, K. J., Boettcher, G. C., Muck, R. E., Weimer, P. J. & Casler, M. D. Harvest and storage of two perennial grasses as biomass feedstocks. *Trans. ASABE* **53**, 359–370 (2010).

[26] Yu, T. E. et al. Influence of particle size and packaging on storage dry matter losses for switchgrass. *Biomass Bioenerg.* **73**, 135–144. 10.1016/j.biombioe.2014.12.009 (2015).

[27] Williams, S. D. & Shinners, K. J. Farm-scale anaerobic storage and aerobic stability of high dry matter sorghum as a biomass feedstock. *Biomass Bioenerg.* **46**, 309–316. 10.1016/j.biombioe.2012.08.010 (2012).

[28] Josse, J. & Husson, F. missMDA: A package for handling missing values in multivariate data analysis. *J. Stat. Softw.* **70**, 1–31 (2016).

[29] Wilson, L. T., Y. Yang, & J. Wang. Integrated agricultural information and management system (iAIMS): World Climatic Data. January 2024. https://beaumont.tamu.edu/ClimaticData/ (Accessed 4/28/2025) (2024).

[30] Nelson, D. L., Cox, M. M. & Hoskin, A. A. *Lehninger principles of biochemistry* 8th edn. (Macmillan Learning, 2021).

[31] Coble, C. G. & Egg, R. Dry matter losses during hay production and storage of sweet sorghum used for methane production. *Biomass* **14**, 209–217 (1987).

[32] Ashbell, G. & Weinberg, Z. G. Top silage losses in horizontal silos. *Can. Agric. Eng.* **34**, 171–175 (1992).

[33] Borreani, G., Tabacco, E., Schmidt, R. J., Holmes, B. J. & Muck, R. E. Silage review: Factors affecting dry matter and quality losses in silages. *J. Dairy Sci.* **101**, 3952–3979. 10.3168/jds.2017-13837 (2018). 29685272 10.3168/jds.2017-13837

[34] Khanchi, A., Jones, C. & Sharma, B. Characteristics and compositional variation in round and square sorghum bales under different storage conditions. ASABE meeting paper no. 096672. St. Joseph, MI: ASABE; 2009. (2009).

[35] Savoie, P., D’Amours, L., Amyot, A. & The´riault, R. Effect of Density, cover, depth, and storage time on dry matter loss of corn silage. ASABE meeting paper no. 061048. St. Joseph, MI, USA: ASABE; 2006. (2006).

[36] Lenz, H., Idler, C., Hartung, E. & Pecenka, R. Open-air storage of fine and coarse wood chips of poplar from short rotation coppice in covered piles. *Biomass Bioenerg.* **83**, 269–277. 10.1016/j.biombioe.2015.09.018 (2015).

[37] Whittaker, C., Yates, N. E., Powers, S. J., Misselbrook, T. & Shield, I. Dry matter losses and greenhouse gas emissions from outside storage of short rotation coppice willow chip. *Bioenergy Res.* **9**, 288–302. 10.1007/s12155-015-9686-y (2016). 27398132 10.1007/s12155-015-9686-y PMC4913936

[38] McGechan, M. B. A review of losses arising during conservation of grass forage: Part 2, storage losses. *J. Agric. Eng. Res.* **45**, 1–30. 10.1016/s0021-8634(05)80135-0 (1990).

[39] McAllister, T. A. & Hristov, A. N. in 18th Annual Western Canadian Dairy Seminar (WCDS). 381–399 (2000).

[40] Rees, D. V. H. A discussion of sources of dry matter loss during the process of haymaking. *J. Agric. Eng. Res.* **27**, 469–479. 10.1016/0021-8634(82)90085-3 (1982).

[41] Graham, S., Eastwick, C., Snape, C. & Quick, W. Degradation of biomass fuels during artificial storage in a laboratory environment. *Int. J. Low-Carbon Technol.* **7**, 113–119. 10.1093/ijlct/cts029 (2012).

[42] Athmanathan, A., Emery, I. R., Kuczek, T. & Mosier, N. S. Impact of temperature, moisture, and storage duration on the chemical composition of switchgrass, corn stover, and sweet sorghum bagasse. *Bioenergy Res.* **8**, 843–856. 10.1007/s12155-014-9563-0 (2015).

[43] Pordesimo, L. O., Hames, B. R., Sokhansanj, S. & Edens, W. C. Variation in corn stover composition and energy content with crop maturity. *Biomass Bioenerg.* **28**, 366–374. 10.1016/j.biombioe.2004.09.003 (2005).

[44] Pordesimo, L. O., Sokhansanj, S. & Edens, C. W. Moisture and yield of corn stover fractions before and after grain maturity. *Trans. ASAE* **47**, 1597–1603. 10.13031/2013.17589 (2004).

[45] Oyedeji, O., Sokhansanj, S. & Webb, E. Spatial analysis of stover moisture content during harvest season in the US. *Trans. Asabe* **60**, 1015–1023. 10.13031/trans.11898 (2017).

[46] Hale, A. L. et al. Registration of “Ho 02–113” Sugarcane. *J. Plant Registr.* **7**, 51–57. 10.3198/jpr2011.11.0605crc (2013).

[47] Gordon, V. S. et al. Registration of “UFCP 84–1047” sugarcane for use as a biofuel feedstock. *J. Plant Registr.* **10**, 251–257. 10.3198/jpr2015.03.0021crc (2016).

[48] Gordon, V. S. et al. Registration of “UFCP 87–0053” sugarcane for use as a biofuel feedstock. *J. Plant Registr.* **10**, 258–264. 10.3198/jpr2015.03.0022crc (2016).

[49] Bera, T. et al. Seasonal growth dynamics and yield potential of biomass sorghum in the Southeastern US. BMC Plant Biology (in press) (2026). 10.1186/s12870-025-08032-1 PMC12882182 41519749

[50] Wilson, L. T., Yang, Y., Dou, F. & Bera, T. Final technical report: sustainable herbaceous energy crop production in the Southeast United States (DE-EE0008522). Texas A&M AgriLife Research and Extension Center, Beaumont, Texas. 135 pp. (2024).

[51] Therasme, O., Volk, T. A., Eisenbies, M. H., San, H. & Usman, N. Hot water extracted and non-extracted willow biomass storage performance: Fuel quality changes and dry matter losses. *Front Energy Res*10.3389/fenrg.2019.00165 (2020).

[52] NREL, N. R. E. L. Biomass compositional analysis laboratory procedures – laboratory analytical procedures ( LAPs) (https://www.nrel.gov/bioenergy/biomass-compositional-analysis) (Accessed 8/28/2025). (2025).

[53] Payne, C. E. & Wolfrum, E. J. Rapid analysis of composition and reactivity in cellulosic biomass feedstocks with near-infrared spectroscopy. *Biotechnol. Biofuels*10.1186/s13068-015-0222-2 (2015). 25834638 10.1186/s13068-015-0222-2 PMC4381445

[54] Sluiter, A. & Wolfrum, E. Near infrared calibration models for pretreated corn stover slurry solids, isolated and in situ. *J. Near Infrared Spectrosc.* **21**, 249–257. 10.1255/jnirs.1065 (2013).

[55] Wolfrum, E. et al. Multivariate calibration models for sorghum composition using near-infrared spectroscopy. Technical Report NREL/TP-510056838. Golden, CO: National renewable energy laboratory (NREL). (2013).

[56] Templeton, D., Scarlata, C., Sluiter, J. & Wolfrum, E. Compositional analysis of lignocellulosic feedstocks. 2 Method Uncertainties. *J. Agricult. Food Chem.* **58**, 9054–9062. 10.1021/jf100807b (2010). 10.1021/jf100807b PMC2923869 20669952

[57] SAS. SAS STAT 13.2 User’s Guide. (SAS Institute, 2015).

[58] Zar, J. H. Biostatistical analysis. (1984).

[59] Yang, Y., Wilson, L. T. & Wang, J. Development of an automated climatic data scraping, filtering and display system. *Comput. Electron. Agric.* **71**, 77–87 (2010).

[60] Schreiber-Gregory, D. N. Multicollinearity: What Is It, Why Should We Care, and How Can It Be Controlled? Paper 1404–2017 (https://support.sas.com/resources/papers/proceedings17/1404-2017.pdf) (Accessed 3/15/2024). SAS Global Forum, April 2–5. Orlando, FL. (2017).

[61] Wicklin, R. Collinearity in regression: The COLLIN option in PROC REG. https://blogs.sas.com/content/iml/2020/01/23/collinearity-regression-collin-option.html (Accessed 3/15/2024) (2020).

[62] R Core Team. The R project for statistical computing (http://www.R-project.org) (Accessed 10/12/2025) (2025).

